# DNA Methylation Analysis of the Macrosatellite Repeat Associated with FSHD Muscular Dystrophy at Single Nucleotide Level

**DOI:** 10.1371/journal.pone.0115278

**Published:** 2014-12-29

**Authors:** Claudia Huichalaf, Stefano Micheloni, Giulia Ferri, Roberta Caccia, Davide Gabellini

**Affiliations:** 1 Dulbecco Telethon Institute at San Raffaele Scientific Institute, Division of Regenerative Medicine, Stem Cells, and Gene Therapy, DIBIT2, 5A3, Via Olgettina 58, 20132, Milano, Italy; 2 Università Vita-Salute San Raffaele, Milano, Italy; INSERM UMR S_910, France

## Abstract

Facioscapulohumeral muscular dystrophy (FSHD) is one of the most common inherited diseases of the skeletal muscle. It is characterized by asymmetric muscle weakness and variable penetrance. FSHD is linked to a reduction in copy number of the D4Z4 3.3 kb macrosatellite repeat, located in 4q35. This causes the epigenetic de-repression of FSHD candidate genes leading to disease. Nevertheless, the molecular mechanism responsible for silencing of FSHD candidate genes in healthy subjects is not fully understood. While a role for DNA methylation has been suggested, so far there is limited information regarding the methylation status of the 325 CpGs contained in each D4Z4 unit. Using a human/rodent monochromosomal hybrid cell line containing a single human chromosome 4, we performed an in depth analysis of DNA methylation for the majority of the CpGs inside D4Z4 at single nucleotide level. We found that D4Z4 is not uniformly methylated and that the level of DNA methylation does not correlate with the density of CpG dinucleotides. Moreover, in several D4Z4 regions characterized by near complete methylation, we found specific unmethylated CpGs. These elements are enriched in transcription factor binding sites that could be involved in muscle-specific D4Z4 activity. Our approach also detected differential methylation among different D4Z4 units, suggesting that the D4Z4 array is a mosaic of euchromatic and heterochromatic domains. Finally, we found that DNA methylation and histone de-acetylation are required to maintain FSHD candidate genes repressed. Taken together, our data underscore new players involved in the epigenetic regulation of the FSHD locus that could be targeted for therapeutic purposes.

## Introduction

FSHD is one of the most prevalent neuromuscular disorders [1]. Usually, patients affected by FSHD start to present symptoms in their teens, and by the second decade of life more than 90% have shown signs of the disease [2]. Nevertheless, presentation of FSHD exhibits a wide range of clinical severity and variable age of onset, even between family members with similar DNA mutation. Notably, monozygotic twin discordance has been reported, thus suggesting a strong epigenetic component in the disease [3], [4].

Two forms of the disease have been described: FSHD1 (MIM 158900) and FSHD2 (MIM 158901). Different from most Mendelian disorders, FSHD1 (accounting for 95% of FSHD cases) is not due to a mutation in a protein-coding gene. Instead, it is linked to reduction in copy number of a 3,3 kb macrosatellite repeat called D4Z4, located in the long arm of human chromosome 4 (4q35). In healthy individuals 11 to 100 D4Z4 repeats are found, while FSHD1 patients carry 1 to 10 units [5]. It is increasingly evident that, in healthy subjects, the high D4Z4 copy number mediates the epigenetic repression of 4q35 genes [6], [7]. On the other hand, the reduction of D4Z4 copy number in FSHD1 patients is associated to altered expression of several 4q35 genes [8]–[18]. Consequently, it has been proposed that the disease is caused by the aberrant expression of one or more 4q35 FSHD candidate genes [5]. FSHD2 is a D4Z4 contraction-independent form of the disease. In 85% of FSHD2 patients the disease is brought about by mutations in the epigenetic regulator SMCHD1 [19].

Several aspects of the FSHD molecular mechanism remain unclear. For example, while its involvement has been suggested [19]–[23], evidence for a direct role of DNA methylation in the repression of FSHD candidate genes has never been provided.

D4Z4 is extremely GC rich (73% as opposed to 42% of the average human genome-wide GC content) [24] and displays an unusually high CpG dinucleotide frequency (10% compared to the average human genome frequency of 1%) [25]. Since each D4Z4 unit contains 325 CpGs and in healthy subjects up to 100 D4Z4 repeats are present, the FSHD locus represents one of the more extended CpG islands of the human genome [5]. For this reason, D4Z4 is an attractive candidate for regulation by DNA methylation, a major mechanism of epigenetic repression occurring predominantly in the context of CpG dinucleotides and carried out by the DNA methyltransferases (DNMTs) protein family [26]. Unfortunately, the study of DNA methylation at 4q35 is complicated by the fact that D4Z4–like sequences are present in several other chromosomes beside chromosome 4 [27]. In particular, as a result of an ancient duplication, the subtelomere of chromosome 10q contains a repeat array with 98% identity to D4Z4 [28]. To investigate specifically DNA methylation at 4q35, all studies so far have used Methyl Sensitive Restriction Analysis (MSRA) [29]. Using this approach, only 3 CpG dinucleotides within the 325 CpGs present in each D4Z4 unit were analyzed. By this approach, it has been shown that D4Z4 is highly methylated in healthy subjects and significantly hypomethylated in FSHD patients [20]–[22], [30], [31]. Recently, using Sodium Bisulfite sequencing [32], 74 CpGs of D4Z4 have been investigated in FSHD2 patients, confirming the D4Z4 hypo-methylation in the disease [20]. While these studies strongly suggest a role for DNA methylation in FSHD, they were severely limited in their coverage of the FSHD locus. Moreover, a functional role for DNA methylation in the control of FSHD candidate genes is still missing.

Here, we characterized a cellular model to selectively study the epigenetic status of the human FSHD locus in 4q35. Using this model, we investigated DNA methylation for the majority of the CpGs inside D4Z4 at single nucleotide level. We found extensive variability in DNA methylation along the D4Z4 repeat, spanning the entire range from 0 to 100 percent methylation and irrespectively of CpG density. Moreover, we discovered opposite methylation patterns for different D4Z4 units of the array. Finally, we found strong cooperation between DNA methylation and histone de-acetylation in the repression of FSHD candidate genes. These results provide novel targets for the development of possible therapeutic approaches aimed at normalizing candidate gene expression to treat FSHD.

## Material and Methods

### Genomic DNA purification and Bisulfite conversion

Cell pellets were carefully resuspended in 300 ul of TE per 1×10^6^ of cells. Genomic DNA (gDNA) was prepared by Proteinase K treatment, followed by phenol-chloroform extraction, ethanol precipitation and RNAse digestion.

Bisulfite conversion was performed using EpiTect kit from Qiagen with the following modifications. 1 ug of gDNA per conversion reaction was used. Bisulfite conversion was performed using a thermal cycler with the following conditions: 99°C for 5 minutes, 60°C for 25 minutes, 99°C for 5 minutes, 60°C for 85 minutes, 99°C for 5 minutes and 60°C for 175 minutes, repeating these cycling conditions twice and hold at the end at 20°C. Converted DNA was then cleaned up according to manufacturer recommendations. Next, 2 ul of bisulfite converted DNA were used in PCR. Primers for bisulfite converted gDNA were designed using Methprimer [33]. CpG dinucleotides were excluded from primer sequence, when possible, in order to avoid bias amplification. Primers are listed in Table 1. PCR reactions were performed according to manufacturer recommendation with GoTaq FlexiDNA (Promega).

**Table 1:** Primers used in Bisulfite sequencing analysis.

Region	Primer	Sequence
B1	Fw	GCGTTCGGGTTTGATATC
B1	Rv	TCCCTACGTCGCTCTATCTT
B2	Fw	GGTTGAGGGTTGGGTTTATAGT
B2	Rv	TACACCCTTCCCTACATATTTCC
B3	Fw	GAAATATGTAGGGAAGGGTGTAAGTT
B3	Rv	TCTTAAATATACCAAACCCTCTCTCC
B4	Fw	CTATTTATGAAGGGGTGGAGTTTGTT
B4	Rv	AAAACCAAATCTAAACCCTAAACTC
B5	Fw	GGAGTTTAGGGTTTAGATTTGGTTT
B5	Rv	AAACCCCCTATAAAAAAACCCC
B6	Fw	AGAGGGGATTTTTTAATTTGTTT
B6	Rv	AAATACCTTACATCTACCCCTACC
B7	Fw	GGTAGGGGTAGATGTAAGGTATTT
B7	Rv	TAACCAACCAAATATTCCCC

PCR products were analyzed in 2% electrophoresis agarose gel. PCR products were cloned into pGEM-T vector (Promega) followed by white/blue screening. At least 30 colonies were analyzed using colony PCR and positive clones were sequenced using SP6 and T7 primers. Sequences were analyzed using QUMA (quantification tool for methylation analysis) [34], with an upper limit of unconverted CpHs of 5% and a lower limit of percentage of converted CpHs of 95%.

### Chromatin ImmunoPrecipitation (ChIP)

Chr4/CHO cells were seeded in 5–10 15 cm dishes. Chromatin was prepared as previously described [6]. Immunoprecipitations were carried out at 4°C overnight with overhead rotation using 50 ul of beads previously bound for 3 hours at 4°C with 10 ug of the following antibodies: rabbit anti-H4 (#62-141-13, Millipore), rabbit anti-H3 (#ab1791, Abcam), rabbit anti-acetyl-Histone H3 (#06-599, Millipore), rabbit anti-acetyl-Histone H4 (#06-866, Millipore), rabbit-anti-Trimethyl-Histone H3 (Lys9) (#17-625, Millipore), rabbit anti-SMC3 (Cohesin complex) (ab3914, Millipore), mouse anti-HP1 gamma (#05-690, Millipore), rabbit anti-HDAC1 (#06-729, Millipore), mouse anti-HDAC2 (ab51832, Abcam), rabbit anti-HDAC3 (ab7030, Abcam), mouse anti-DNMT1 (IMG-261A, Imgenex), mouse anti-DNMT3A (IMG-268A, Imgenex), mouse anti-DNMT3B (IMG184A, Imgenex), whole molecule mouse IgG (015-000-003, Jackson Immunoresearch) and whole molecule rabbit IgG (011-000-003, Jackson Immunoresearch). Immunoprecipitated chromatin was washed as previously described [6]. DNA was purified with QIAquick PCR Purification Kit (Qiagen) according to manufacturer recommendations. DNA was analyzed by qPCRs with SYBR GreenER qPCR SuperMix Universal (Life technologies) using Biorad's CFX96 Real-time System.

The specificity of the amplified products was monitored by performing melting curves at the end of each amplification reaction and by verifying the size of the amplification products by agarose gel electrophoresis. The efficiency of each primer was assessed by performing primer validation qPCRs with serial dilutions of reference gDNA or cDNA according to the analysis. Only primers with a 95–105% of efficiency were selected.

Further characterization for primers amplifying regions inside D4Z4 was made by performing in parallel qPCR from a panel of monochromosomal hybrid lines containing each human chromosome. Melting curve and cycle threshold (CT) for each primer was analyzed in order to discriminate chromosomal specificity. Validated primers are listed in Table 2.

**Table 2:** Primers used in ChIP experiments.

Region	Primer	Sequence
p13E11	Fw	TGGGCATTTTCTCATTAGCC
p13E11	Rv	CTGGAGCAGAGATGACCACA
D1	Fw	GAGAGAGGAACGGGAGACCT
D1	Rv	GGACGCTGACCGTTTTCC
D2	Fw	GGAGGCGTGATTTTGGTTT
D2	Rv	GTGGGGAGTCTGCAGTGTG
D3	Fw	TCAGCCGGACTGTGCACTGCGGC
D3	Rv	AGGCCTCGACGCCCTGGGTC
D4	Fw	CCGCGTCCGTCCGTGAAA
D4	Rv	TCCGTCGCCGTCCTCGTC
D5	Fw	TGAGAAGGATCGCTTTCCAG
D5	Rv	CCCTTCGATTCTGAAACCAG
*Beta actin* Promoter	Fw	GCCCATGGGTCTTTGTCTAA
*Beta actin* Promoter	Rv	CCCAATACCCAGCCATAGAG

To normalize for the amount of D4Z4 repeats present, ChIP-qPCR signals were normalized for the input signal of five different single copy regions.

### Mammalian cell culture

Human chromosome 4/CHO (Chr4/CHO) hybrid cell (GM10115) was obtained from the Coriell Institute for Medical Research. Cells were cultured in DMEM-HIGH (Dulbecco's Modified Eagle's Medium, High Glucose with Sodium Pyruvate and L-Glutamine; EuroClone) supplemented with 10% FBS (Foetal Bovine Serum; EuroClone), 1% Penicillin/Streptomycin (100 U/ml final concentration; EuroClone) and Proline (final concentration 0.2 mM; Sigma). Cells were cultured at 37°C in a 5% CO2 humidified incubator.

### HDACs and DNMTs inhibitors Treatment

For 5-Aza-2-deoxycytidine (AZA, Sigma) and Trichostatin A (TSA, Invivogen) treatment, Chr4/CHO cells were seeded at low confluence (about 20%) in growth medium. Cells were treated with AZA only, TSA only or a combination of AZA plus TSA. For the AZA treatment, 24 hours after plating AZA was added to the medium at a final concentration of 1 uM. Media was replaced every day adding 1 uM of fresh AZA for 72 hours. For TSA treatment 1 uM of TSA was added to the media for 12 hours before harvesting. In the case of the combined treatment, cells were seeded at low confluence, next day AZA was added to the media at a final concentration of 1 uM. Media was replaced every day adding 1 uM of fresh AZA for 72 hours. TSA was added to the medium (final concentration 1 uM,) at 60 hours of AZA treatment and cells were collected after 12 hours in order to obtain a final treatment of 72 hours with AZA and 12 hours with TSA.

### Methyl DNA Immunoprecipitation (MeDIP)

For MeDIP analysis, gDNA was purified as previously described. gDNA was fragmented (200–400 bp) per 5 cycles 30 seconds ON 30 second OFF at low intensity with Bioruptor sonication device (Diagenode). gDNA was denatured for 10 minutes in boiling water and immediately cool down on ice for 10 minutes. 500–1000 ng of sonicated gDNA was precipitated following protocol described in [35].

### RNA extraction, retrotranscription and qPCR analysis

Total RNA from cells was extracted using PureLink RNA Mini Kit (Life Technologies). DNAse treatment was performed twice, including one on-column treatment with DNase I (Life Technologies) and a final treatment with TURBO DNase (Life Technologies). cDNA was synthesized according to manufacturer's instructions. Up to 1 ug of RNA was synthesized using SuperScript III First-Strand Synthesis Super-Mix (Life Technologies). qPCRs were performed with SYBR GreenER qPCR SuperMix Universal (Life technologies) using Biorad's CFX96. Relative quantification was calculated with CFX Manager Software V.1.6. Primers used for gene expression are listed in Table 3.

**Table 3:** Primers used in RT-qPCR for gene expression analysis.

Gene	Primer	Sequence
*Gapdh*	Fw	TCAAGAAGGTGGTGAAGCAGG
*Gapdh*	Rv	ACCAGGAAATGAGCTTGACAAA
*Beta-Actin*	Fw	CCCTGAAGTACCCCATTGAA
*Beta-Actin*	Rv	GGGGTGTTGAAGGTCTCAAA
*FRG1*	Fw	TCTACAGAGACGTAGGCTGTCA
*FRG1*	Rv	CTTGAGCACGAGCTTGGTAG
*FRG2*	Fw	ACAAAGGCAAGGATCGGAGC
*FRG2*	Rv	CTGACATAGCTCGCACAGAA
*ANT1*	Fw	GCACATTTTTGTGAGCTGGA
*ANT1*	Rv	CTTGGCTCCTTCGTCTTTTG
*DUX4*	Fw	GCGCAACCTCTCCTAGAAAC
*DUX4*	Rv	AGCAGAGCCCGGTATTCTTC
*DBE-T*	Fw	TCAGCCGGACTGTGCACTGCGG
*DBE-T*	Rv	AGGCCTCGACGCCCTGGGT
*Dnmt1*	Fw	CGCTGCCTGGTCCGCAT
*Dnmt1*	Rv	AACTTCTTGTCATCCACCAC
*Dnmt3a*	Fw	GTCATCCACCAAGACACAATTC
*Dnmt3a*	Rv	GGGGTGTTGAAGGTCTCAAA
*Dnmt3b*	Fw	TGGAGCCAGGACAGCCAGCA
*Dnmt3b*	Rv	CCACCATGGCAGGCCACCAG
*Hdac1*	Fw	CTACTACGACGGGGATGTTG
*Hdac1*	Rv	CGGATTCGGTGAGGCTTC
*Hdac2*	Fw	TAAATCCAAGGACAACAGTGG
*Hdac2*	Rv	GGTGAGACTGTCAAATTCAGG
*Hdac3*	Fw	CGCCTGGCATTGACCCATAG
*Hdac3*	Rv	CTCTTGGTGAAGCCTTGCATA

## Results

### A cellular model to selectively study the epigenetic status of the FSHD locus

**Figure 1:**
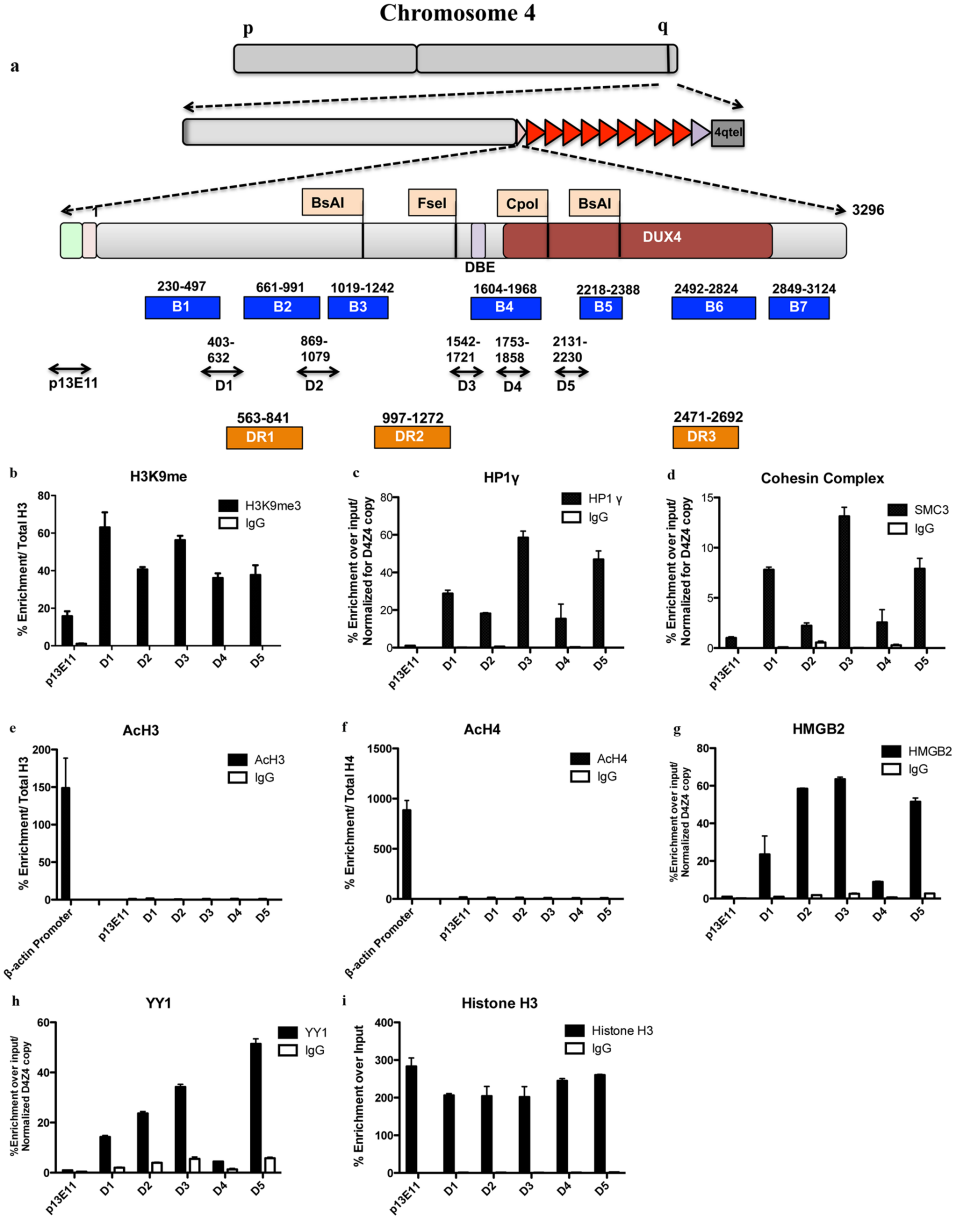
Chr4/CHO cells recapitulate the epigenetic status present at the FSHD locus in healthy subjects. ChIP survey for repressive and actives marks as well as enzymes present inside D4Z4. Recruitment was analyzed by ChIP followed by qPCR with primers located throughout D4Z4. Primers located in the D4Z4 proximal region p13E-11 were used as control. Results are normalized for D4Z4 copy number. **a.** Set of primers spanning D4Z4 used in qPCR and Bisulfite Studies, including location of DR primers from Hartweck et al and Methylation sensitive sites previously reported in literature. **b.** ChIP for H3K9me3. **c.** ChIP for HP1 gamma. **d.** ChIP for Cohesin. **e** and **f.** ChIP for pan acetylated histones H3 and Histone H4 compared with the highly transcribed gene *beta*-*Actin* used as control. **g.** ChIP for HMGB2. **h.** ChIP for YY1. **i.** ChIP for Total Histone H3 showing similar enrichment for all the regions. Error bars correspond to SEM. Panel b, e and f are normalized for the signal of total histone H3. Panel c and d, signal is normalized for D4Z4 copy number as described in Materials and Methods.

The human genome contains sequences homologous to D4Z4 on several chromosomes in addition to 4 [27]. This severely limits the possibility to obtain detailed information of the specific epigenetic status of the 4q35 region. Since D4Z4 is a primate-specific repeat [36], we exploited a commercially available human/rodent monochromosomal hybrid cell line containing, in a Chinese Hamster Ovary (CHO) background, a single human chromosome 4 with 27 D4Z4 units (located on a 4qB subtelomere) derived from a healthy subject (Chr4/CHO) [6], [37]. Considering that no much information regarding the epigenetics background of CHO cells is currently available, we decided, as a first step, to determine if the key epigenetic features of the FSHD locus in healthy subjects are maintained in Chr4/CHO cells. To this purpose, we performed a Chromatin ImmunoPrecipitation (ChIP) survey of proteins and histone marks previously described at the FSHD locus in healthy subjects [6], [7], [12]. Immunopurified material was analyzed by real-time quantitative PCR (qPCR) using several primer pairs spanning D4Z4 (Fig. 1a). One additional primer pair located in a region immediately proximal to the repeat array (p13E-11) was used as control (Fig. 1a). H3K9me3, HP1gamma and SMC3 (Cohesin Complex) were found enriched at D4Z4 in healthy subjects and significantly decreased in FSHD patients [7]. Nevertheless, this analysis has been conducted by qPCR with just one primer pair (Q-PCR in [7] and D4 in our analysis). While we confirmed that H3K9me3, HP1gamma and Cohesin are all enriched over D4Z4 with respect to the region immediately proximal to the repeat array, we also found that they display different enrichment patterns along the repeat (Fig. 1b–1d). H3K9me3 is enriched at similar levels in all D4Z4 regions analyzed (Fig. 1b). On the contrary, HP1gamma and Cohesin display a higher enrichment in regions D1, D3 and D5 (Fig. 1c–d). These results are in agreement with previous findings suggesting that the H3K9me3 density is not sufficient to recruit HP1gamma and Cohesin to D4Z4. Indeed, it has been shown that an additional factor, which may be expressed in a cell type-specific manner, is required for HP1gamma and Cohesin binding to D4Z4 and that they depend on each other for their recruitment to D4Z4 [7].

H3K27me3 was reported to be enriched at D4Z4 in healthy subjects and significantly decreased in FSHD patients [6], [12] and previous work done by our group has shown that the pattern of H3K27me3 is maintained in Chr4/CHO cells [6].

In healthy subjects, D4Z4 displays low histone H4 acetylation levels [38]. Accordingly, we found that in Chr4/CHO cells the D4Z4 repeat array and the region immediately proximal to it displayed very low histone H3 and H4 acetylation levels compared to the promoter region of the transcriptionally active gene betaActin (Fig. 1e–f).

Notably, YY1 and HMGB2, two other proteins involved in the regulation of FSHD candidate genes [17], are enriched over D4Z4 but with a different distribution compared to H3K9me3, HP1gamma and Cohesin (Fig. 1g and h). Moreover, similar enrichment between the different D4Z4 regions was observed for total Histone H3 (Fig. 1i), supporting the specificity of the above results.

Finally, FISH analyses have shown a distribution restricted to the nuclear periphery for 4q35 [39], [40] and this property is conserved in Chr4/CHO cells [6].

Overall, these results indicate that Chr4/CHO cells faithfully recapitulates the epigenetic features of the FSHD locus in healthy subjects allowing for a high-resolution analysis of it.

### Variable DNA methylation inside D4Z4

Using Chr4/CHO cells, we designed a strategy to examine global average methylation levels within 7 different D4Z4 regions, at single nucleotide level. Allowing us to cover the majority (219 out of 325) of the CpG dinucleotides located inside each repeat unit. Purified gDNA was treated with bisulfite, which converts unmethylated cytosine to uracil but does not convert methylated cytosine. Bisulfite converted genomic DNA was amplified with bisulfite-converted primers and PCR products were cloned and sequenced. The sequence of at least 10 independent clones from 3 independent bisulfite converted genomic DNA preparations was analyzed using QUMA (quantification tool for methylation analysis; [34]) software, setting a 95% cutoff for conversion in order to minimize the effect of incomplete conversion in the analysis.

**Figure 2:**
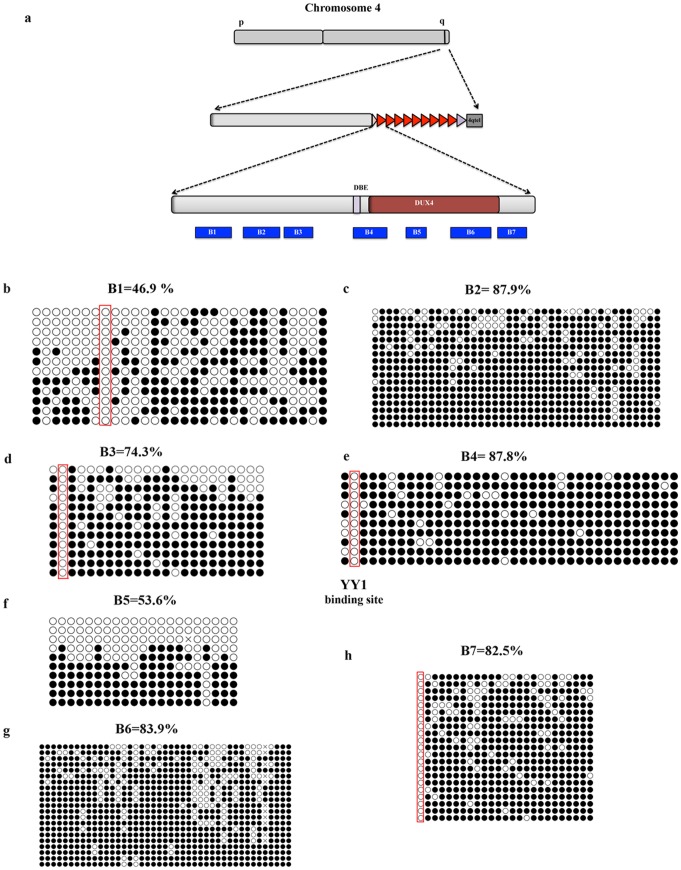
D4Z4 methylation analysis by bisulfite sequencing. At least 30 clones were analyzed by Quantification Tool for Methylation Analysis (QUMA). The figure represents cumulative results for ≥10 independents clones sequenced in both directions. **a.** Scheme representing bisulfite primer location. **b.** Region B1 contains 30 CpGs dinucleotides. **c.** Region B2 contains 41 CpGs dinucleotides. **d.** Region B3 contains 23 CpGs dinucleotides. **e.** Region B4 contains 36 CpGs dinucleotides. **f.** Region B5 contains 21 CpGs dinucleotides. **g.** Region B6 contains 43 CpGs dinucleotides. **h.** Region B7 contains 25 CpGs dinucleotides. Red boxes highlight single non-methylated CpGs. Open Circle correspond to unmethylated CpG. Close circle correspond to methylated CpG dinucleotide.

Among seven D4Z4 regions analyzed (Fig. 2a), region B1 displays the lowest methylation level (average 46.9% and a range from 0% to 91.7%) despite its 30 CpG dinucleotides and high percentage of CpG dinucleotides (Figs. 2b and S1). B1 is located 45 nucleotides upstream of a previously analyzed region (DR1) displaying similar average level of DNA methylation (55%) in healthy subjects [20] and contains two CTCF binding sites previously described by Ottaviani et al [41]. Interestingly, CTCF binding to 4q35 displays an inverse correlation to D4Z4 copy number [41].

Region B2 contains 41 CpGs and displays the highest level of methylation among the regions analyzed with an average methylation of 87,9%, ranging from 42 to 100% (Figs. 2c and S1). On the other hand, region B3 spans 23 CpGs and has an average methylation of 74.3% ranging from 0% to 100% (Figs. 2d and S1). This region contains a BsaAI restriction site previously surveyed by MSRA [22], [23], [30] and bisulfite sequencing [20] and was shown to be highly methylated. Consistent with previous reports, the BsaAI site displays a 100% level of methylation in Chr4/CHO cells (Fig. 2d).

Region B4 contains 36 CpGs with the second highest average methylation (87,8%) among the regions analyzed (Figs. 2e and S1). Inside B4 is present the D4Z4 Binding Element (DBE), previously shown to be bound by a repressor complex composed by YY1, HMGB2 and nucleolin [17]. The consensus-binding site of YY1 contains one CpG dinucleotide and DNA methylation inhibits YY1 DNA binding *in vitro* and *in vivo* [42]. Despite the fact that region B4 displays a very high methylation on average, we found that the YY1 consensus inside DBE [17] is unmethylated (Fig. 2e) providing a possible molecular explanation for the ability of YY1 to bind DBE and repress FSHD candidate genes in healthy subjects [17].

For region B5, we found an unexpected result (Fig. 2f). While the average methylation level of its 21 CpG dinucleotides is 53.6%, among the sequenced clones we found either non-methylated or highly methylated clones (Figs. 2f and S1). In this particular region it was previously reported enrichment for the euchromatic histone mark H3K4m2 and the heterochromatic histone marks H3K9me3 and H3K27me3 [7]. Moreover, ChIP-re-ChIP experiments showed that H3K9me3 co-exists with H3K27me3 but never with H3K4me2 [7]. Taken together our bisulfite sequencing and previous ChIP-re-ChIP results strongly suggest that, at least in region B5, the D4Z4 array is a mosaic with some heterochromatic and some euchromatic repeat unit.

Region B6 contains 49 CpGs and displays methylation of 85.5% on average, ranging from 50% to 100% (Fig. 2g). This region corresponds to a similar fragment analyzed by Hartweck in FSHD2 patients (called DR3 see Fig. 1a) and displays a comparable methylation level [20].

Finally, region B7 is highly methylated at levels similar to B6. This region contains 25 CpG dinucleotides and displays 82.5% of average methylation ranging from 0% to 100% (Figs. 2h and S1).

Overall, our bisulfite sequencing analyses indicate a high degree of methylation inside D4Z4, consistent with previous reports [20], [22], [23], [30]. Nevertheless, we obtained several unexpected findings. In the different regions analyzed, we found highly variable methylation levels (spanning the entire range from 0 to 100%) that do not correlate with the density in CpGs. This suggests that, contrary to our expectation, D4Z4 is not a single uniformly repressive domain. Moreover, we found isolated, non-methylated CpGs embedded inside regions of highly methylated CpGs. It is tempting to speculate that these sites could represent entry points for DNA binding proteins relevant for the biology of D4Z4. Our analysis also supports the notion that in region B5 the D4Z4 array could be a mosaic with some D4Z4 unit displaying a euchromatic environment and other D4Z4 units presenting a heterochromatic environment.

### DNMTs and HDACs are selectively recruited to the FSHD locus

DNA methylation is carried out by members of the DNA methyltransferase (DNMT) family [26]. There are three enzymatically active members of the family: Dnmt1, Dnmt3a and Dnmt3b. To determine which DNMT is responsible for DNA methylation at D4Z4, we performed ChIP using antibodies specific for Dnmt1, Dnmt3a or Dnmt3b. Immunopurified material was analyzed by qPCR using the same primer pairs of Fig. 1a.

**Figure 3:**
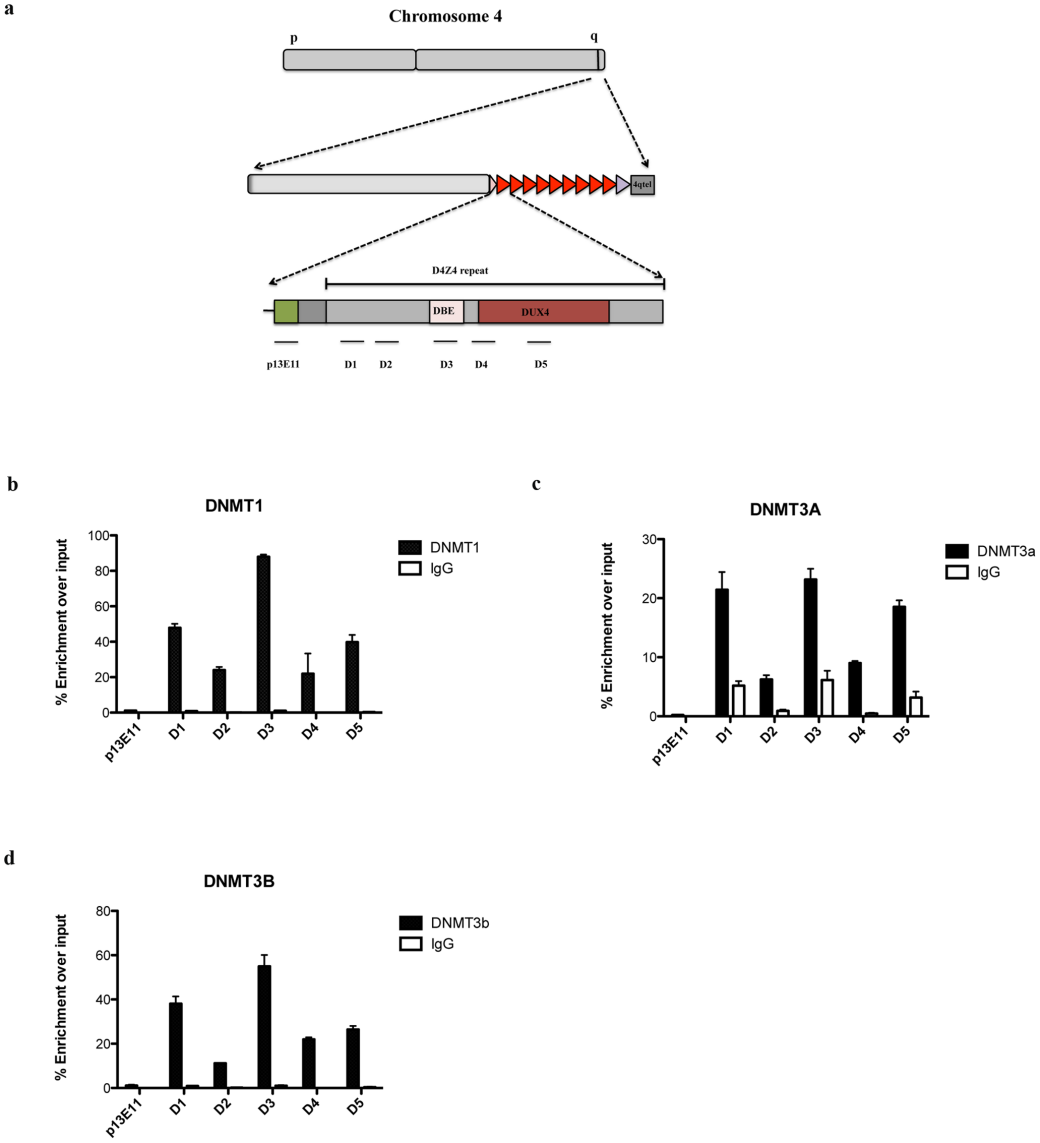
DNMTs are enriched to the FSHD locus. ChIP survey for Dnmt1, Dnmt3A and Dnmt3B in Chr4/CHO cells. Recruitment was analyzed by qPCR with primers located throughout D4Z4. Primers located in the D4Z4 proximal region p13E-11 were used as control. **a.** Scheme of primers used in qPCR. **b.** ChIP results for Dnmt1. **c.** ChIP results for Dnmt3a. **d.** ChIP results for Dnmt3b. Error bars correspond to SEM. Panel b through d signal was normalized for D4Z4 copy number as described in Material and Methods.

Although they are expressed at very different levels in Chr4/CHO cells (S2 Fig.), all three DNMTs were specifically enriched inside D4Z4 compared to the region immediately proximal to the repeat array (Fig. 3b–d). Dnmt1 was the enzyme of the family displaying the highest enrichment (Fig. 3b), followed by Dnmt3b and then Dnmt3a (Figs. 3c and 3d). Surprisingly, each DNMT displayed a different enrichment in the various D4Z4 regions analyzed (Fig. 3). Moreover, the patterns of enrichment were not correlated with the CpG density or the DNA methylation level of the different regions (compare Figs. 1a and 3).

**Figure 4:**
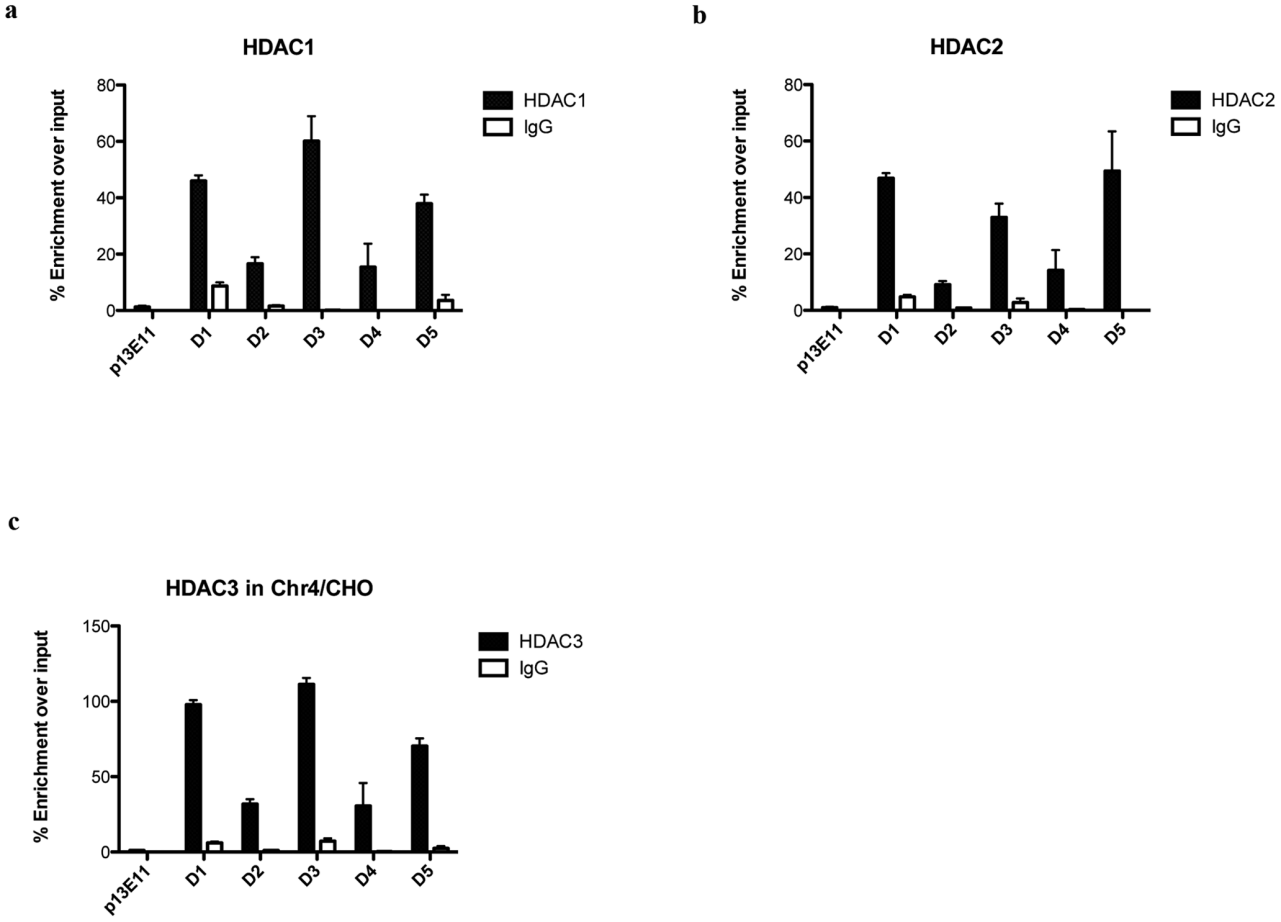
HDACs are enriched to the FSHD locus. ChIP survey for Hdac1, Hdac2 and Hdac3 in Chr4/CHO cells. Recruitment was analyzed by qPCR with primers located throughout D4Z4. Primers located in the D4Z4 proximal region p13E-11 were used as control. **a.** ChIP for Hdac1. **b.** ChIP for Hdac2. **c.** ChIP for Hdac3. Error bars correspond to SEM. Panel a through c signal was normalized for D4Z4 copy number as described in Material and Methods.

Given the hypoacetylation level found inside D4Z4 in Chr4/CHO cells (Fig. 1e–f), we decided to investigate the association of histones deacetylases (HDACs) to the FSHD locus. HDACs are divided in 4 classes, with class I being the most abundant and ubiquitously expressed [43]. For this reason, we investigated the association of class I HDACs to D4Z4. We surveyed by ChIP, the presence of Hdac1, Hdac2 and Hdac3 on D4Z4 in Chr4/CHO cells. Following this approach, we found that all three HDACs were selectively enriched inside D4Z4 as compared to the control region immediately proximal to the repeat array (Fig. 4). Similar to DNMTs, HDACs displayed variable enrichment levels in the various D4Z4 regions analyzed (Fig. 4a–c). Interestingly, Hdac3 was the enzyme showing the strongest enrichment inside D4Z4 (Fig. 4c) even if in CHO/Chr4 cells it is expressed at much lower levels compared to Hdac1 and 2 (S3 Fig.).

Collectively, theses results suggest that the three DNMTs and HDACs analyzed could redundantly collaborate in maintaining the FSHD locus repressed.

### Role of DNA methylation and histone de-acetylation in FSHD candidate gene expression

**Figure 5:**
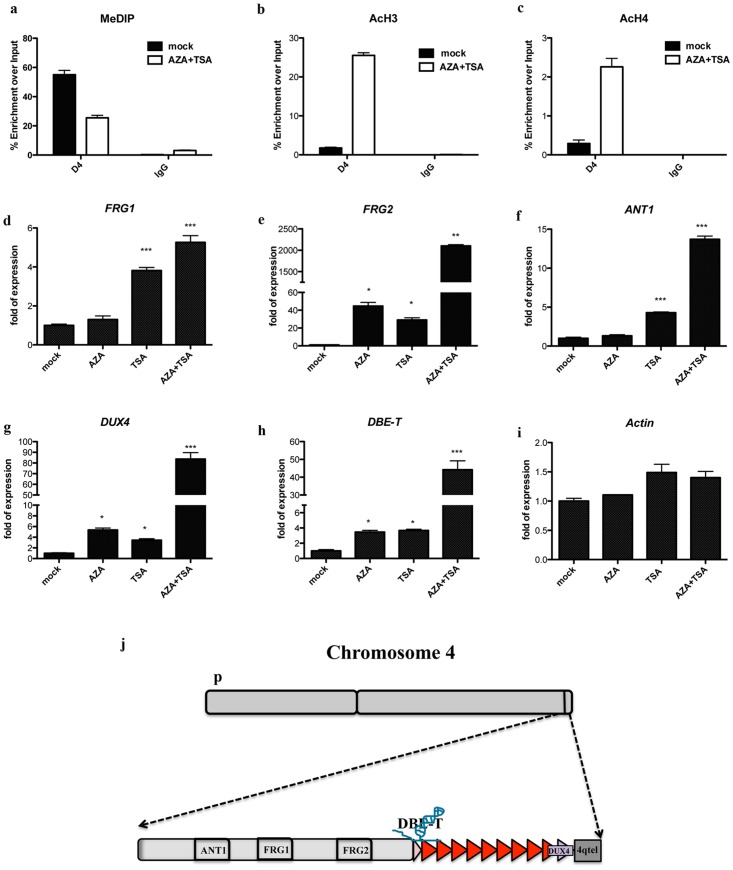
Effect of DNMTs and HDACs inhibitors on the expression of FSHD candidate genes. Expression analyses for 4q35 genes upon single treatment with AZA, TSA or a combined treatment with AZA plus TSA. Relative expression of 4q35 genes, using GAPDH as normalizator and mock as reference. Graphs represent average of three independent experiments. Error bar correspond to SEM. Statistic analysis was performed using Graphpad Software. Samples were analyzed by One way ANOVA followed by Dunnett's Multiple Comparison Test. * P≤0.05; ** p≤0.01; *** p≤0.001. **a.** MeDIP inside D4Z4 showing reduction in DNA methylation upon AZA + TSA treatment. **b.** ChIP showing reduced acH3 in AZA + TSA treated cells. **c.** ChIP showing reduced acH4 in AZA + TSA treated cells. **d.** *FRG1* expression. **e.** *FRG2* expression analysis. **f.** *ANT1* expression analysis. **g.** *DUX4* expression analysis. **h.** LncRNA *DBE-T* expression analysis. **i.** *Beta*-*Actin* expression analysis, as control. **j.** Scheme showing location of genes of interest in the 4q35 region.

DNA methylation and histone de-acetylation are commonly associated to negative regulation of gene expression [26], [43]. Since loss of D4Z4 repeats leads to de-repression of FSHD candidate genes located on the 4q35 region [44], we hypothesized a direct role for DNMTs and HDACs in the transcriptional regulation of the FSHD region. To assess it, we first investigated the effect of downregulating the expression of DNMTs or HDACs by RNAi. Unfortunately, single depletion of the DNMTs or HDACs associated to D4Z4 was not sufficient to cause a significant alteration of FSHD candidate gene expression (data not shown); further supporting their redundant activity at 4q35. To test this hypothesis, we used small molecule inhibitors to obtain a specific but general ablation of either DNMTs or HDACs activity. Specifically, we used 5′-Aza-2′deoxycytidine (AZA) also know as Decitabine, a chemical analogue of cytosine which is incorporated into DNA during DNA replication [45]. This leads to a rapid loss of DNA methylation, due to the formation of adducts with DNMTs and loss of their activity [45]. We also used Trichostatin A (TSA), an organic compound that selectively inhibits HDACs of class I and II but not class III [46]. TSA fit into the catalytic site of HDACs, a tubular structure with a zinc atom at its base [47], blocking the catalytic reaction by chelating this atom with its hydroxamic acid moiety [47]. Methyl DNA Immunoprecipitation (MeDIP) and ChIP with anti-acetylated histone H3 and H4 were used to confirm loss of DNA methylation and increase of histone acetylation at D4Z4 following AZA and TSA treatment (Fig. 5a, b and c).

While the lncRNA *DBE-T* and the two protein-coding genes most closely located to D4Z4 (*DUX4* and *FRG2*) were mildly up-regulated upon AZA treatment alone, more distantly located 4q35 protein-coding genes (*FRG1* and *ANT1*) were unaffected (Fig. 5d–h). On the contrary, all 4q35 genes analyzed were moderately de-repressed by TSA alone treatment (Fig. 5d–h). Interestingly, the combination of AZA plus TSA treatments resulted in a significant and synergic effect on the expression of all FSHD candidate genes (Fig. 5d–h). Importantly, no significant change was observed for the control gene *beta-Actin*, treatments on 4q35 genes (Fig. 5i).

Altogether, our results strongly suggest that DNA methylation and histone de-acetylation are required to keep FSHD candidate genes repressed.

## Discussion

### A model system to focus epigenetic studies selectively on 4q35 D4Z4 repeats

Due to the presence of sequences closely related to D4Z4 on almost all acrocentric chromosomes [48], in human cells it is very difficult to perform a detailed analysis of the FSHD locus. To overcome this limitation, we took advantage of the primate-specificity of the D4Z4 repeat [36]. Thus, we used a rodent/human monochromosomal hybrid containing one human chromosome 4 derived from a healthy donor (Chr4/CHO) as the only human counterpart. This way, we were able to focus our analysis specifically on the D4Z4 array located on the human chromosome 4.

The first step of our analysis was to investigate the epigenetic features displayed by the D4Z4 array in Chr4/CHO cells [7], [12], [38], [49]. The histone mark H3K9me3 is usually associated with heterochromatin and in healthy subjects it is enriched on the FSHD locus where it was proposed to be the result of SUV39H1 histone methyltransferase activity [7]. Accordingly, RNAi mediated knockdown of SUV39H1 translated into a reduction in H3K9me3 in HeLa cells [7]. Heterochromatin binding protein HP1 is usually associated to transcriptional silencing and is recruited by methylated H3K9 [50]. HP1 gamma binds to D4Z4 in healthy subjects and its enrichment at D4Z4 is reduced following H3K9me3 loss in FSHD patients [7]. In Chr4/CHO cells, we found a selective enrichment on D4Z4 for H3K9me3, HP1gamma and Cohesin (protein found associated to D4Z4 [7]) (Fig. 1). Moreover, inside D4Z4 we found distinct and variable enrichment patterns for the above histone mark and proteins (Fig. 1 b–d) and for other FSHD candidate gene regulator like YY1 and HMGB2 (Fig. 1g and h). Future studies will be required to elucidate the functional relevance of these findings.

Gene repression is also associated to low levels of histone acetylation [51]. Accordingly, hypo-acetylation of histone H4 has been reported for D4Z4 in healthy subjects [38]. In agreement, we confirmed histone H4 hypo-acetylation at the level of the D4Z4 repeat array in Chr4/CHO cells and extended similar results to histone H3 (Fig. 1e–f). Therefore, together with previous results [6], our data indicates that Chr4/CHO cells faithfully recapitulate a number of epigenetic features previously described at the endogenous FSHD locus in healthy subjects. Nevertheless, we cannot exclude that certain aspects of the D4Z4 epigenetic landscape may vary in different species or cell types.

### Non-uniform DNA methylation inside D4Z4

Until now, analyses of DNA methylation at the FSHD locus have been conducted mainly using the traditional method of restriction digestion of gDNA with enzymes differentially sensitive to cytosine methylation (MSRA), followed by Southern blotting. Unfortunately, this approach presents several limitations as: the need of high amounts of gDNA, the requirement of specific probes for Southern analysis and the availability of informative restriction sites inside the region of interest. The above limitations and the presence of multiple D4Z4-like repeats in the human genome restricted the analysis of DNA methylation at D4Z4 by MSRA to only 3 out of the 325 CpGs inside the repeat [21]–[23], [30]. Recently, an additional study used bisulfite sequencing but investigated only 74 CpGs in FSHD2 patients [20]. Instead, by combining Chr4/CHO cells and bisulfite sequencing, we were able to analyze 219 out of the 325 CpG dinucleotides located inside each D4Z4 unit. While confirming D4Z4 hypermethylation, we found that DNA methylation is not uniformly distributed inside the repeats and that it does not correlate with the density of CpGs. For example, we found that region B1 displays a very low methylation level on average compared to the other regions analyzed (Figs. 2 and S1). This suggests the existence of mechanisms that could shield from methylation regions of functional relevance. ChIP experiments indicate that all three main DNMTs (Dnmt1, Dnmt3A and Dnmt3b) are specifically enriched at D4Z4 (Fig. 2), consistent with a redundant role for these enzymes at D4Z4. Since we did not found any significant correlation between the profile of DNMTs enrichment and the level of DNA methylation, it seems unlikely that the differential methylation of different D4Z4 regions is due to a differential recruitment of specific DNMTs. On the other end, nucleosome positioning could influence DNA methylation patterns throughout the genome [52] and DNMTs appear to have nucleosome-bound DNA as a preferential target [52]. Hence, a relevant issue to be investigated in the future is whether the differential methylation inside D4Z4 correlates with the location of nucleosomes inside the repeat.

Another interesting finding from our bisulfite sequencing analysis is the identification of four individual CpGs (regions B1, B3, B4 and B7) that are never methylated even if they are surrounded by CpGs that are methylated in 100% of the clones analyzed (Fig. 2). It has been previously reported that transcription factor binding causes reduced methylation at the actual binding motif [53]. Accordingly, we found a single CpG dinucleotide with 0% methylation inside region B4 that corresponds to the binding site of the transcription factor YY1. YY1 is required for repression of FSHD candidate genes in healthy subjects [17] and its DNA binding is inhibited by DNA methylation [54], [55]. Based on this, it is tempting to speculate that the single unmethylated CpGs inside regions B1, B3 and B7 underscore binding sites for yet to be discovered DNA binding proteins regulating the activity of D4Z4. This justifies future studies correlating transcription factors recruitment to D4Z4 with its epigenetic status.

CpG methylation at promoter regions has been traditionally considered a potent silencing mechanism that blocks binding of transcription factors. On the contrary, it has recently been shown that several transcription factors recognize selectively methylated sequences or sequences different from their known consensus sequence when the DNA is methylated, at least in vitro [56]. Therefore, a number of sequence-specific DNA binding proteins may bind and regulate the activity of different D4Z4 units according to their DNA methylation levels.

Beside single non-methylated CpGs, we also found evidence of differentially methylated D4Z4 repeat units. The various D4Z4 units of the array are nearly identical. There are few SNPs that allow distinguishing the first, most proximal unit from internal units, however they are erased upon bisulfite conversion. Thus, the information obtained by bisulfite sequencing is an “average” of the DNA methylation status for the different D4Z4 units of the array. Interestingly, for region B5, bisulfite sequencing identified either non-methylated or fully methylated clones. Considering that in bisulfite sequencing several independent PCR clones are sequenced, the black or white results for region B5 could belong to different D4Z4 units. This suggests the co-existence of hypo- and hyper-methylated D4Z4 units, at least for the region covered by primers B5. Moreover, ChIP-re-ChIP experiments with anti-H3K4me2 and anti-H3K9me3 antibodies also identified heterogeneity in the different D4Z4 units [7]. Collectively, these results strongly suggest that euchromatic units coexist side by side with heterochromatic units inside the D4Z4 array.

*DUX4*, the leading FSHD candidate gene, displays two main alternative splicing isoforms [13]. *DUX4* short is not pathogenic, while *DUX4* full-length is associated with FSHD [13], [37], [57]. In addition, there is evidence that *DUX4* alternative splicing could be epigenetically regulated [10]. Whether the differential methylation of D4Z4 bears any functional relevance for the regulation of *DUX4* alternative splicing remains to be determined.

### DNA methylation and histone de-acetylation are required to maintain FSHD candidate genes repressed

While several studies associated D4Z4 hypomethylation to FSHD [7], [20], [23], [30], [41], [49], direct evidence for a role of DNA methylation in the control of FSHD candidate gene expression is lacking. Using small molecules inhibitors of DNMTs, we found that inhibition of DNA methylation causes a mild de-repression of the 4q35 protein-coding genes *FRG2* and *DUX4*, and the lncRNA *DBE-T* (Fig. 5). Interestingly, these genes are located very close or inside the D4Z4 array (Fig. 5j). Therefore, the reduction of DNA methylation could directly induce a change in the tridimensional structure of D4Z4 locus, allowing a more open chromatin state, and thus facilitating the expression of these genes.

Similar to DNA methylation, histone de-acetylation is a repressive mechanism commonly used by cells to shutdown gene expression [43]. We found that Hdac1, Hdac2 and Hdac3 are specifically enriched to the D4Z4 region, consistent with its low acetylation in healthy subjects (Figs. 1e–f and 4a–c). Similar to DNMTs, HDACs data suggested a redundant role of the three enzymes at D4Z4. Nevertheless, our results indicate that histone de-acetylation is required to maintain the lncRNA *DBE-T* and FSHD candidate genes repressed (Fig. 5).

**Figure 6:**
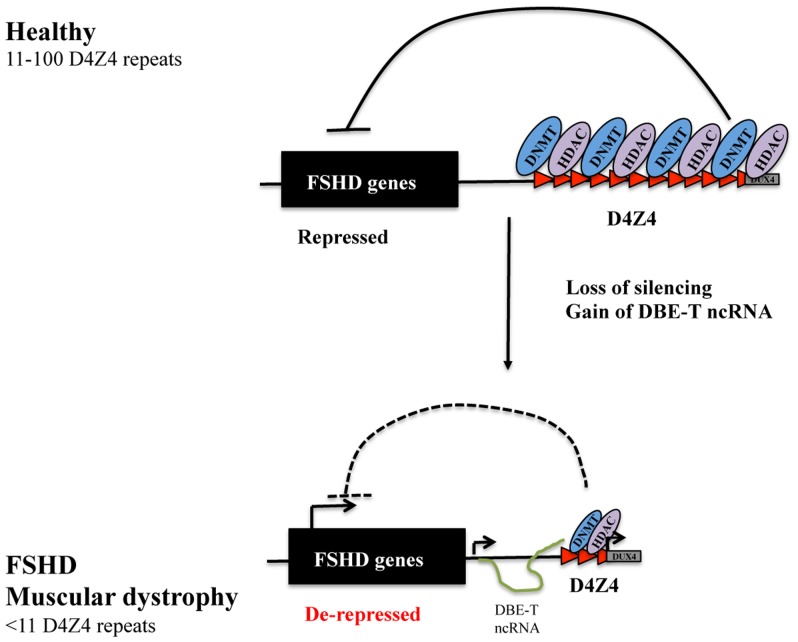
Model for DNMTs and HDACs contribution in 4q35 gene repression. Healthy individuals carrying from 11 to 100 D4Z4 units display a high enrichment for DNMTs and HDACs, redundantly contributing to the repressive environment of the FSHD locus. Deletion of D4Z4 units is associated to reduced DNMTs and HDACs, leading to de-repression of several FSHD candidate genes in FSHD.

A significant and synergic effect on 4q35 gene expression was observed upon combined DNMTs and HDACs inhibition. This suggests that these two families of proteins may collaborate and mutually influence themself in the regulation of the FSHD locus (Fig. 6). Accordingly, several studies indicate that specific histone modifications serve as marks that are used by HDACs and histone methyltransferases to recruit DNMTs and subsequently target DNA methylation to specific chromatin domains especially in *de novo* methylation in early development [58], [59]. On the other hand, DNA methylation may also influence the maintenance of histone modifications through cell division, since it has been proposed that methylcytosine-binding proteins such as methyl-CpG binding protein 2 (MeCP2) recruit HDACs to methylated regions [60], [61].

## Conclusions

This work was focused on elucidating the role of DNA methylation and histone deacetylation on the control of the epigenetic status of D4Z4 and the expression of FSHD candidate genes. Through the use of bisulfite sequencing, we were able to provide single nucleotide information of the methylation status for most of D4Z4 CpGs dinucleotides. While confirming previous results, we also found that DNA methylation is non-homogeneous inside D4Z4. In particular, we identified CpGs that are never methylated located inside a context of fully methylated CpGs and found evidence that the D4Z4 repeat array could be a mixture of euchromatic and heterochromatic repeats. These features could be involved in differential recruitment of regulatory factors to the FSHD region and in the regulation of *DUX4* alternative splicing. Altogether, our studies elucidate additional layers in the regulation of the epigenetic landscape of the FSHD locus and the expression of 4q35 genes identifying novel potential therapeutic targets.

## Supporting Information

S1 Fig **Primer location and average methylation levels.** **a.** Bisulfite Primer location. **b** Average percentage of methylation along D4Z4 by bisulfite sequencing analysis.
(TIF)

S2 Fig **DNMTs expression in Chr4/CHO cells.** DNMT transcripts levels in Chr4/CHO cells by RT-qPCR. Results are expressed as relative expression over *Gapdh*.
(TIF)

S3 Fig **HDACs expression in Chr4/CHO cells.** HDAC transcripts levels in Chr4/CHO cells by RT-qPCR. Results are expressed as relative expression over *Gapdh*.
(TIF)
