# Correction: DNA Methylation Analysis of the Macrosatellite Repeat Associated with FSHD Muscular Dystrophy at Single Nucleotide Level

**DOI:** 10.1371/journal.pone.0119742

**Published:** 2015-03-20

**Authors:** The PLOS ONE Staff

The following information is missing from the Funding section: Additionally, this study was supported by the Italian Telethon Foundation (https://www.telethon.it/en/). Davide Gabellini is a Dulbecco Telethon Institute Senior Scientist.

The complete, correct funding information is as follows: This work was supported by the Association Française contre les Myopathies (http://www.afm-telethon.com/); the ERA-Net for Research on Rare Diseases (http://www.erare.eu/); the European Research Council (http://erc.europa.eu/); the Italian Epigenomics Flagship Project (http://www.epigen.it/); the Italian Ministry of Health (http://www.salute.gov.it/) and the FSHD Global Research Foundation (http://fshdglobal.org/). Additionally, this study was supported by the Italian Telethon Foundation (https://www.telethon.it/en/). Davide Gabellini is a Dulbecco Telethon Institute Senior Scientist.
