# Supplementary figures and images for: DNA Methylation Analysis of the Macrosatellite Repeat Associated with FSHD Muscular Dystrophy at Single Nucleotide Level
Source: PLoS One. 2014 Dec 29;9(12):e115278. doi: 10.1371/journal.pone.0115278 (PMC4278900; doi:10.1371/journal.pone.0115278)

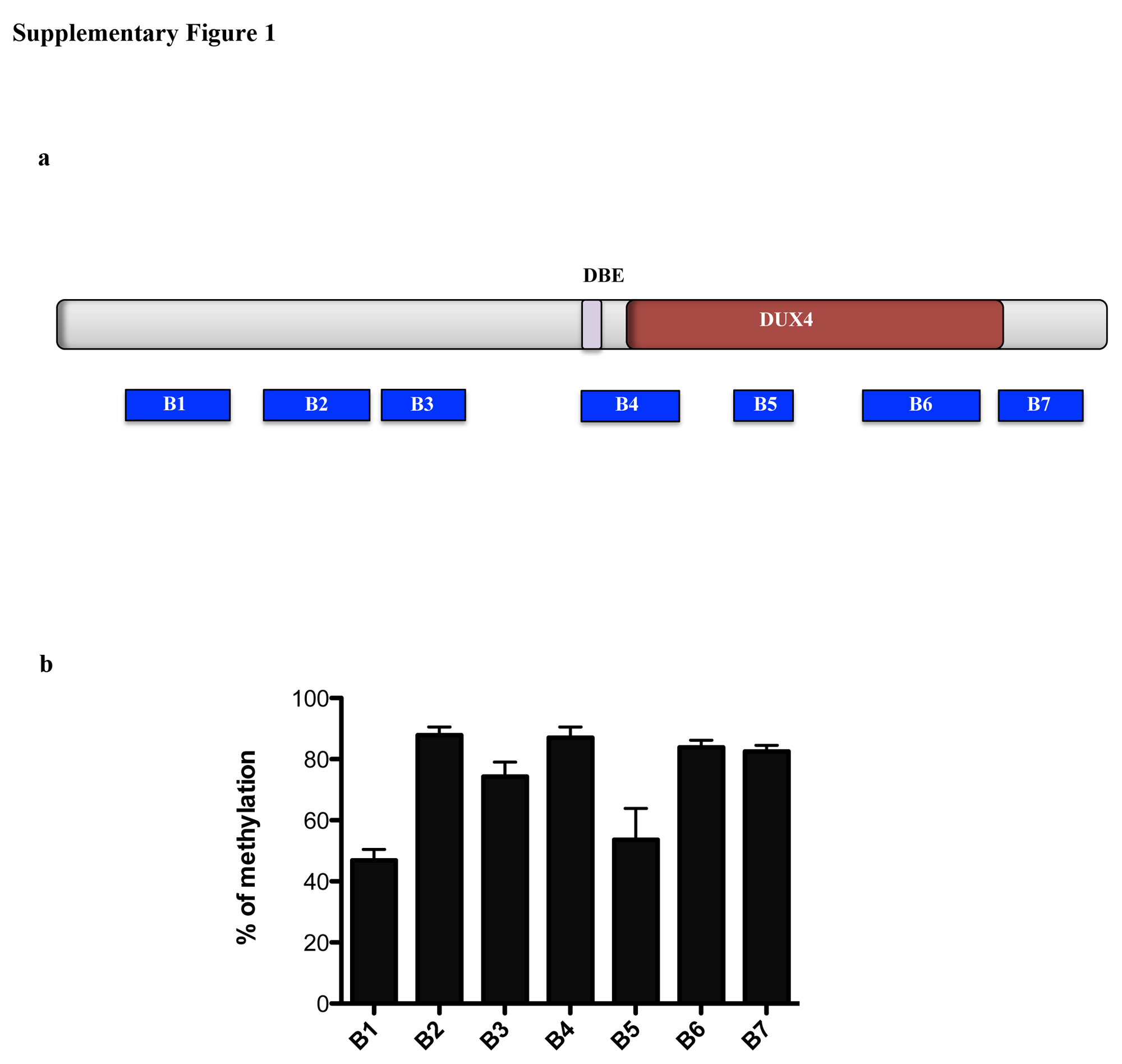

Supplement: S1 Fig — Primer location and average methylation levels. a. Bisulfite Primer location. b Average percentage of methylation along D4Z4 by bisulfite sequencing analysis. (TIF) [file pone.0115278.s001.tif]

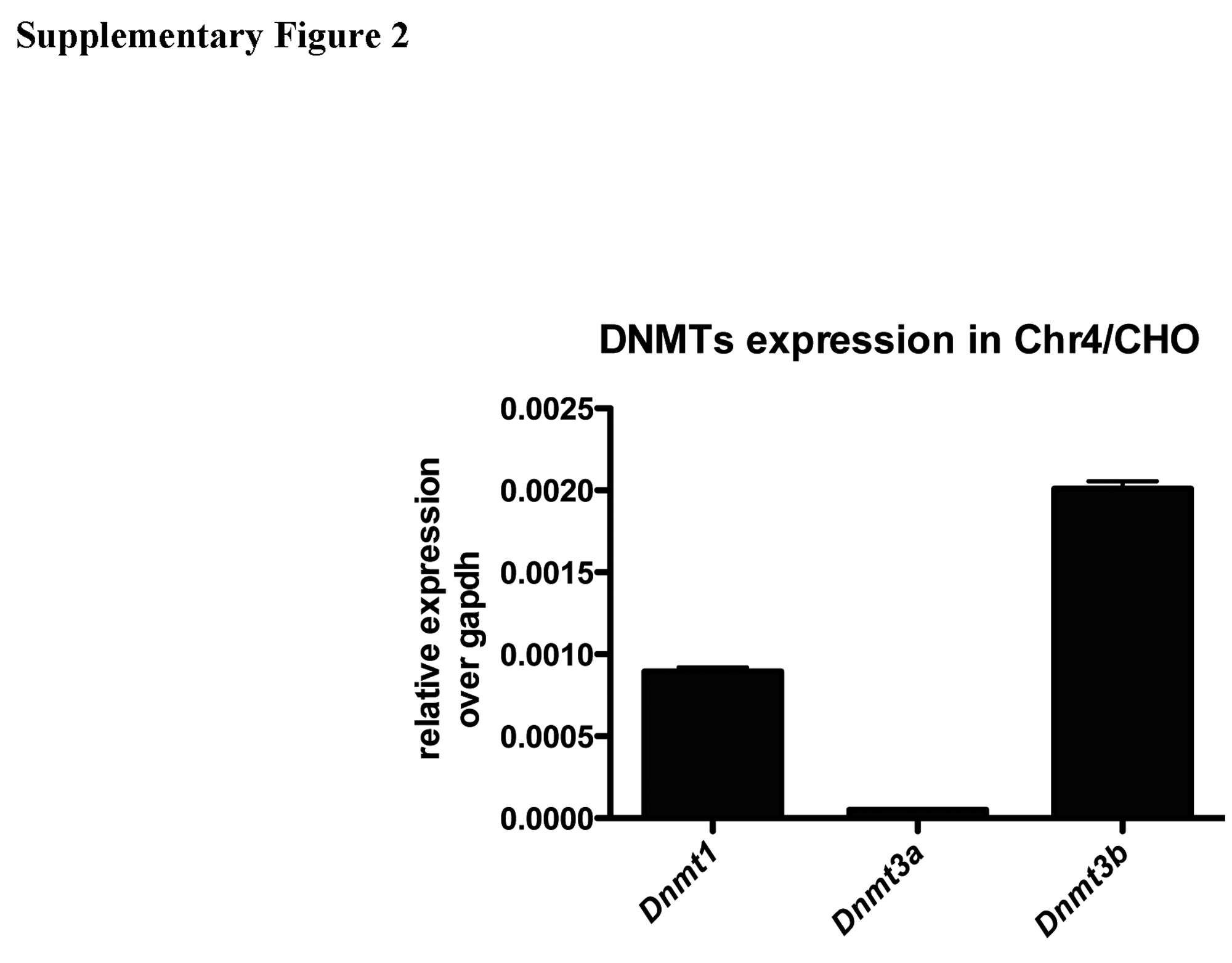

Supplement: S2 Fig — DNMTs expression in Chr4/CHO cells. DNMT transcripts levels in Chr4/CHO cells by RT-qPCR. Results are expressed as relative expression over Gapdh. (TIF) [file pone.0115278.s002.tif]

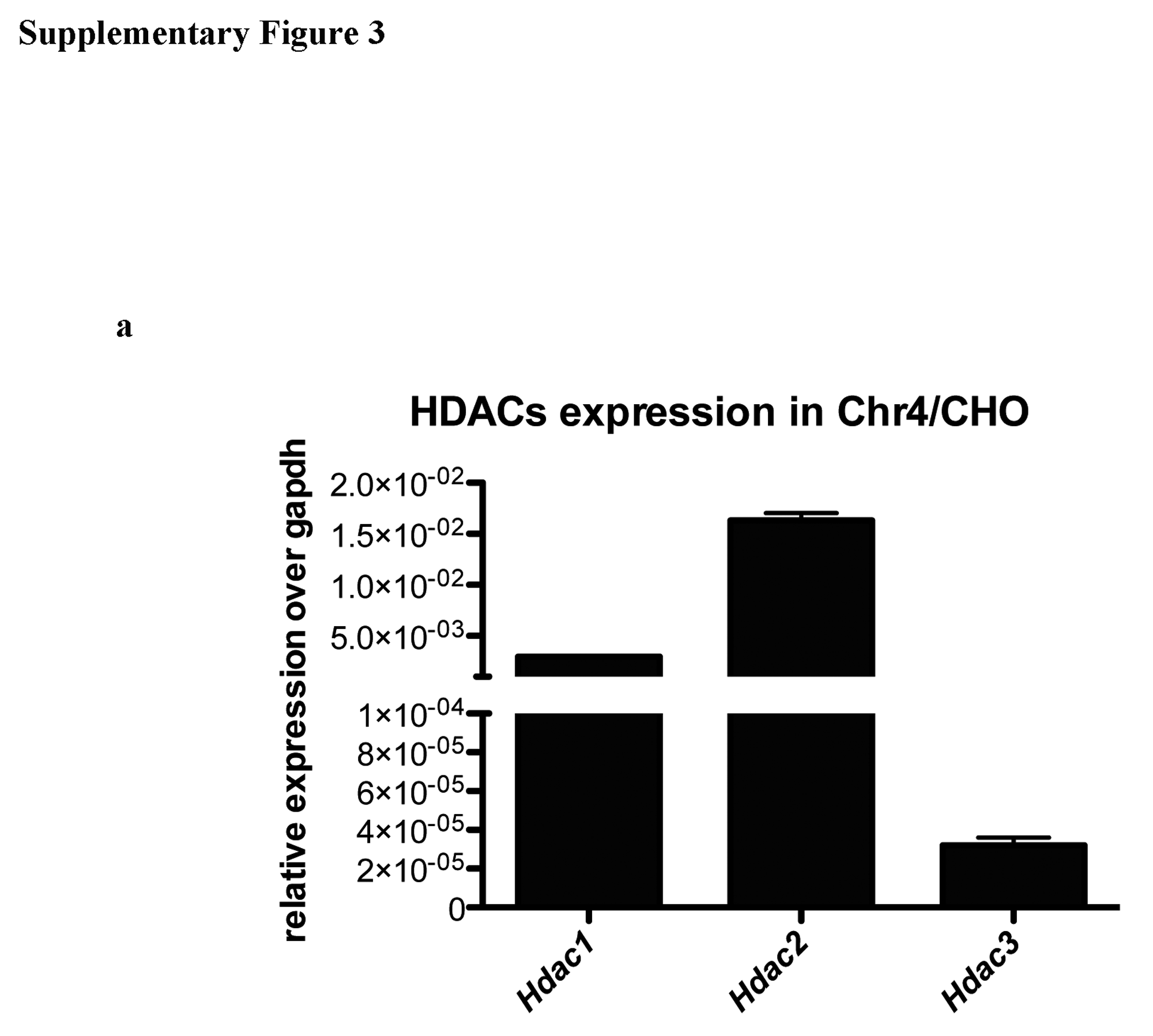

Supplement: S3 Fig — HDACs expression in Chr4/CHO cells. HDAC transcripts levels in Chr4/CHO cells by RT-qPCR. Results are expressed as relative expression over Gapdh. (TIF) [file pone.0115278.s003.tif]
